# Magnetic Hardening: Unveiling Magnetization Dynamics in Soft Magnetic Fe–Ni–B–Nb Thin Films at Cryogenic Temperatures

**DOI:** 10.3390/nano14141218

**Published:** 2024-07-18

**Authors:** Ansar Masood, Liubov Belova, Valter Ström

**Affiliations:** Department of Materials Science and Engineering, KTH-The Royal Institute of Technology, Brinellvägen 23, 100 44 Stockholm, Sweden; ansarm@kth.se (A.M.); valter@kth.se (V.S.)

**Keywords:** low-temperature magnetization, magnetic hardening, heterogeneous amorphous films, spin-glass, magnetic transitions

## Abstract

Recent advancements in amorphous materials have opened new avenues for exploring unusual magnetic phenomena at the sub-nanometer scale. We investigate the phenomenon of low-temperature “magnetic hardening” in heterogeneous amorphous Fe–Ni–B–Nb thin films, revealing a complex interplay between microstructure and magnetism. Magnetization hysteresis measurements at cryogenic temperatures show a significant increase in coercivity (*H_C_*) below 25 K, challenging the conventional Random Anisotropy Model (RAM) in predicting magnetic responses at cryogenic temperatures. Heterogeneous films demonstrate a distinct behavior in field-cooled and zero-field-cooled temperature-dependent magnetizations at low temperatures, characterized by strong irreversibility. This suggests spin-glass-like features at low temperatures, which are attributed to exchange frustration in disordered interfacial regions. These regions hinder direct exchange coupling between magnetic entities, leading to magnetic hardening. This study enhances the understanding of how microstructural intricacies impact magnetic dynamics in heterogeneous amorphous thin films at cryogenic temperatures.

## 1. Introduction

Amorphous thin films, lacking long-range ordered atomic structures, present a captivating sphere for exploration, offering significant challenges and opportunities [1]. Particularly intriguing are their soft magnetic properties, given the absence of long-range ordered magnetism, which can be envisioned for potential applications extending to Magnetics-on-Silicon technologies, including microinductors for high-frequency power conversion [2]. These metastable materials, typically fabricated through physical vapor deposition (PVD), exhibit a homogeneously dispersed disordered atomic structure [1]. However, at the sub-nanometer scale, these films unveil complex structural irregularities, encompassing agglomerated chemical phases, pre-existing nucleation sites, and fluctuating intensities of atomic disorder [3,4]. Magnetization reversal in amorphous films is profoundly shaped by these sub-nanometer scale structural deviations [5]. For example, in Fe–Zr systems, small changes in the level of amorphization can lead to significant transformations in magnetic behavior, ranging from superparamagnetism to spin-glass-like characteristics [6]. The development of these structural anomalies in amorphous films depends on multiple factors, including alloy composition (such as the enthalpy of mixing and atomic size mismatch), fabrication techniques (like pulsed laser deposition and magnetron sputtering), and deposition parameters (such as thermal budget, the energy and angle of landing atoms, and deposition pressure) [3,4,6]. Accurately gauging these structural complexities in amorphous systems presents challenges, given the limitations of state-of-the-art structural analysis methodologies in detecting compositional differences and irregularities in short-range ordered structures at the sub-nanometer scale [4,5]. However, a precise expedition into these structural intricacies holds the potential to unravel the fundamental mechanisms defining unconventional magnetization reversals, paving the way for innovative technological advancements.

Exploration into magnetization reversals takes on an intensified interest within hetrogeneous amorphous materials [3,4,7], notably within the domain of two-phase nanocrystalline materials formed by the controlled annealing of amorphous metal precursors [8,9,10,11,12,13,14,15]. In these materials, nanometer-sized crystallites coexist within an amorphous matrix, offering a unique avenue for investigating interparticle interactions and examining the influence of nanocrystallite size, volume fraction, and the role of the intergranular matrix phase in shaping magnetization reversal behaviors [11,12,13,14,15]. The extended Random Anisotropy Model (RAM) has been instrumental in explaining intergranular exchange interactions in these materials, taking into account magnetic and structural parameters, such as exchange- and structural-correlation lengths [11]. It effectively demonstrates how the magnetocrystalline anisotropy of nanometric grains is averaged out, resulting in exceptional soft magnetic properties at room temperature [11]. Additionally, it accounts for magnetic hardening during the early stages of the nucleation process when nanocrystallites are widely separated [13], as well as near the Curie point of the intergranular amorphous phase [16], due to weak intergranular exchange interactions.

However, the RAM model encounters limitations in predicting temperature-dependent magnetizagion reversal behaviors at cryogenic temperatures, such as magnetic hardening or softening. This complexity amplifies when amorphous materials introduce sub-nanometer scale anomalies, stemming from various factors such as a low glass-forming ability (GFA) of the alloy system [17], the nature of the fabrication method [3,4], and the associated thermal budget [3,7]. Additionaly, challenges in accurately identifying the phases of crystallites and their corresponding volume fractions further pose a formidable challenge in precisely understanding magnetization reversal at cryogenic temperatures, thereby adding layers of complexity to the predictive capabilities of existing models [3].

Amorphous thin films have rarely showcased instances of magnetic hardening at cryogenic temperatures, which is an attribute largely ascribed to their distinctive feature of the absence of grains and grain boundaries. In the scope of this investigation, we unveil a captivating manifestation of magnetic hardening within heterogeneous amorphous thin films at cryogenic temperatures. We attribute this phenomenon to the intriguing spin-glass-like behavior of the nanometric anomalies embedded in the amorphous matrix, which is a feature not explained by the conventional RAM model. This study enhances our understanding of magnetization reversal behaviors in complex hetrogeneous amorphous thin-film systems at cryogenic temperatures.

## 2. Experimental

Thin films were deposited onto a Si substrate using pulsed laser deposition (PLD), employing a Nd-YAG laser, (Continuum NY 81C-10, λ = 355 nm). Surface morphology of the films was investigated by scanning electron microscopy (SEM) and focused ion beam (FIB, Nova 600 NanoLab by FEI Co.) methodology. The crystal structure was scrutinized via X-ray diffraction (XRD) with Cu-Kα radiation, (Siemens D5000, λ = 1.54 Å). A magneto-thermo-gravimetry (Perkin Elmer) technique was utilized to investigate the structural anomalies embedded in the amorphous matrix. In-plane magnetic hysteresis loops were measured using a Superconducting Quantum Interference Device, (SQUID, Quantum Design, MPMS2) magnetometer, encompassing a temperature range from liquid helium to room temperature at a maximum field of 10 kOe. The zero-field-cooled (ZFC) and field-cooled (FC) temperature-dependent magnetization responses, *M*(*T*), were measured at various probe fields (1–10 Oe) using the SQUID system.

## 3. Results and Discussion

Analysis of the film’s surface morphology revealed a homogeneously dispersed disordered atomic structure, lacking the nanocolumnar structures typically observed in PVD-based amorphous films [18]. This conforms to the characteristic feature of conventional amorphous alloys, which is distinct from nanostructured amorphous films known as “nanoglass” [19], thereby confirming the absence of nanometric amorphous grains or grain boundaries. Microstructural analysis via XRD did not reveal discernible Bragg’s peaks, indicating an X-ray amorphous structure of films. However, magneto-thermo-gravimetry (MTG) uncovered sub-nanometer-scale magnetic entities embedded heterogeneously within the amorphous matrix. These entities were identified as sub-nanometer-scale *α*-Fe embedded in a ferromagnetic amorphous matrix with a Curie point ((*T_C_*)*_am_*) = 225 K [4]. The presence of these magnetic entities within the amorphous matrix was attributed to primary nucleation seeds of the crystallites. The expected agglomeration of Fe nuclei was anticipated due to the low GFA of the Fe–Ni–B–Nb alloy [17,20], the thin film fabrication process [3], and the thermal budget during deposition [7]. Further details on thin film deposition and structural investigations have been published elsewhere [4].

**Figure 1:**
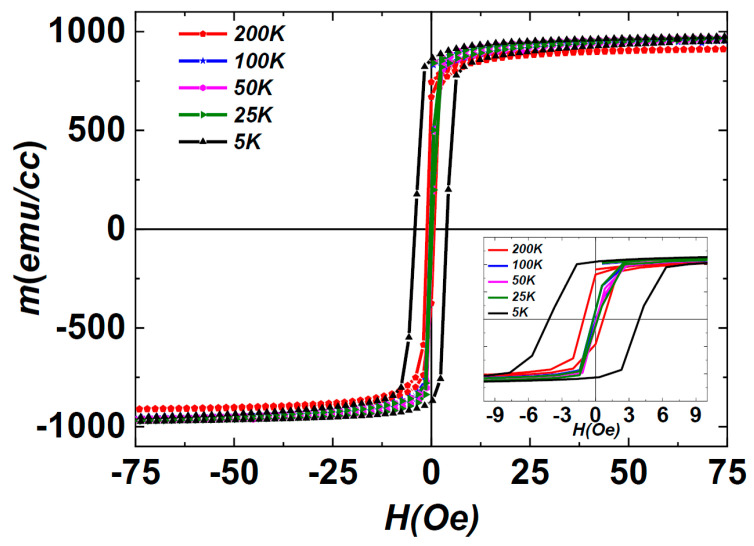
Magnetic hysteresis loops, *M*(*H*), illustrating in-plane magnetization for the 408 nm heterogeneous amorphous film over a temperature range from 5 K to 200 K.

**Figure 2:**
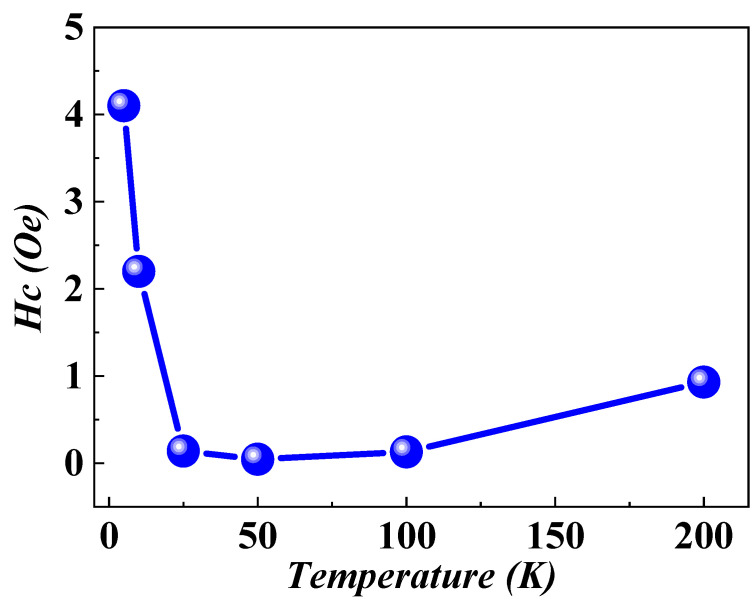
The temperature-dependent modulation of the coercivity (*H_C_*) determined through in-plane magnetic hysteresis loops (*M*(*H*)). The *H_C_* was measured after achieving saturation magnetization by applying a maximum field of 10,000 Oe.

In the investigation of temperature-dependent magnetization, 408 nm film was subjected to a cooling process from 200 K to 5 K in the absence of an external magnetic field. Subsequently, in-plane magnetization loops, *M*(*H*), were measured at various temperatures ranging from 5 K to 200 K, as illustrated in Figure 1. The coercivity (*H_C_*) of the films was initially recorded as 0.93 Oe at 200 K, gradually decreasing to its minimum of 0.05 Oe at 50 K. However, below 25 K, a remarkable change in *H_C_*, referred to as magnetic hardening, was observed, showing a continuous increase to 4.1 Oe at 5 K. This temperature-dependent behaviour of *H_C_*, illustrated in Figure 2, underscores a dynamic transformation in the magnetic state of the system with evolving temperature.

The ultra-low *H_C_* at 0.05 Oe and the formation of a simple square hysteresis loop within the temperature range of 25 K to 200 K exemplify characteristic behaviours observed in soft magnetic amorphous and nanocrystalline alloys. The Curie point of the amorphous matrix was determined as 225 K [4]. Below the Curie temperature of 225 K, the ferromagnetic nature of the amorphous structure enables the exchange coupling of Fe nuclei, resulting in a high *M_R_*/*M_S_* (0.8) ratio indicative of well-coordinated magnetic moments, thereby enhancing the ultra-soft magnetic characteristics of the films. However, a noteworthy deviation from the anticipated smooth behaviour emerges with the observed increase in *H_C_* at temperatures ≤ 25 K, which is acknowledged as magnetic hardening. This unexpected phenomenon challenges conventional expectations of *H_C_* behaviour in heterogeneous amorphous films at low temperatures, suggesting the presence of intricate mechanisms. The intricacies of this magnetic hardening, particularly at cryogenic temperatures, introduce a compelling avenue for exploration within the framework of existing models of soft magnetic amorphous materials.

The *H_C_* observed in amorphous and nanocrystalline materials can be explicated through the Random Anisotropy Model (RAM) initially offered by Alben [21] for monolithic amorphous alloys and subsequently modified by Herzer [11] for nanocrystalline materials. According to the single-phase RAM model, *H_C_* is contingent upon the ratio of particle size (*d*) to exchange-correlation length (*L_ex_*), where *L_ex_* = (*A*/*K*)^1/2^, with ‘*K*’ and ‘*A*’ representing the anisotropy and exchange stiffness constants, respectively. In the context of amorphous materials, the RAM model postulates that local magnetic anisotropy is randomly distributed, contributing to an effective anisotropy (*K_eff_*) that diminishes over a broader scale, thereby resulting in the ultra-low *H_C_* observed in amorphous alloys [21]. Conventionally, at low temperatures, one anticipates a monotonic rise in *H_C_* for amorphous alloys due to pronounced magnetostriction and the dominant contributions of magnetoelastic interactions and domain wall pinning by voids or defects [3,22]. However, our investigation reveals a significant increase in *H_C_* below 25 K, suggesting that the magnetic entity embedded in the amorphous matrix (i.e., Fe-nuclei) predominantly dictates the mechanisms of magnetization reversal in these films. The atomic structure of the films under study exhibits complexity, resembling two-phase materials with sub-nanometer nuclei dispersed within the amorphous matrix, thereby challenging direct application of the RAM model [3].

To reveal the role of Fe-nuclei in these heterogeneous films, we evaluated an extended RAM model of two-phase nanocrystalline materials, accounting for relevant magnetic parameters such as exchange stiffness and anisotropy constants, as well as structural factors like volumetric ratios and grain size, for both the crystalline phase and amorphous matrix, as proposed by Herzer [11]. According to the extended model, exchange coupling between Fe-nuclei is facilitated through the amorphous matrix, with its magnetic state being crucial in determining magnetic softness [3]. The extended RAM model has been successful in interpreting magnetic hardening in heterogeneous nanocrystalline materials, particularly during primary crystallization [13] and near the Curie temperature of the amorphous matrix [16], where surprisingly weak intergranular exchange interactions are anticipated [3].

However, in the current scenario, at temperatures substantially below the Curie point of the amorphous matrix (i.e., *T_C(am)_* = 225 K), where Fe-nuclei are well-exchange-coupled, material parameters such as magnetic anisotropy and the exchange constant demonstrate weak temperature dependence [10]. Consequently, no dramatic changes in *H_C_* or magnetic hardening are expected where intergranular interactions exhibit notably strong exchange coupling [3,22]. This suggests the presence of additional participating factors beyond direct exchange coupling through the amorphous matrix committing to magnetic hardening observed at liquid-helium temperatures. These complexities of magnetic hardening at cryogenic temperatures challenge existing models of soft magnetic materials, thereby necessitating further investigation [3].

**Figure 3:**
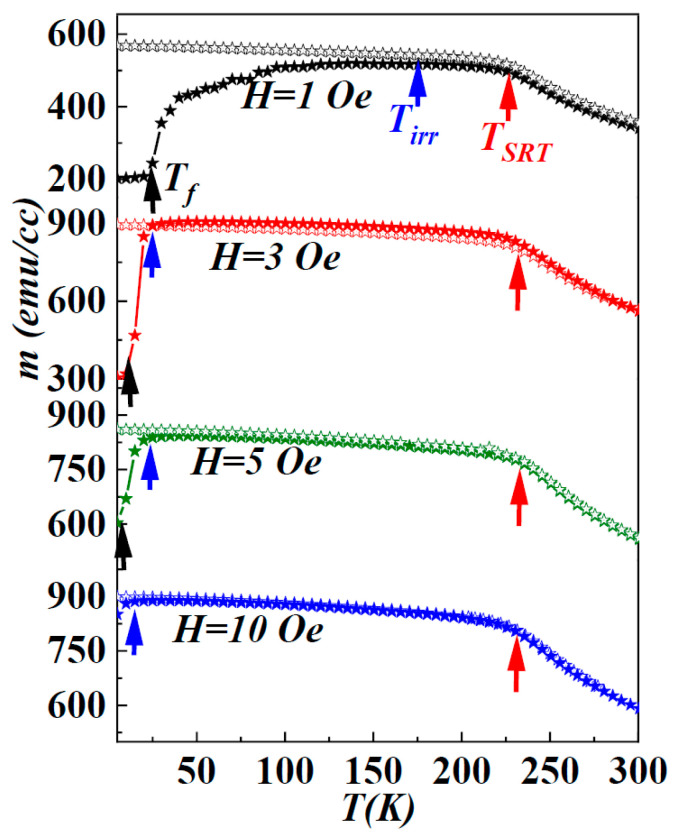
Temperature dependence of magnetization (*M*(*T*)) for the 408 nm film under various probe fields (1–10 Oe); illustrated with solid symbols for zero-field-cooled (ZFC) and hollow symbols for field-cooled (FC) states.

To explore the intricacies of infinitesimal Fe-nuclei embedded within an amorphous matrix, and their profound impact on the magnetization reversal process, particularly the phenomenon of magnetic hardening at cryogenic temperatures, a comprehensive examination of temperature-dependent magnetization (*M*(*T*)) was conducted. This examination was scrutinized under various probe fields ranging from 1 to 10 Oe, in both zero-field-cooled (ZFC) and field-cooled (FC) states. The results unveil intricate insights into the film’s magnetic dynamics, as illustrated in Figure 3. The observed evolution of distinct magnetization states unfolds over the temperature range of 5 K to 300 K, with notable emphasis on three critical magnetic transition temperatures—*T_f_* (spin-freezing temperature), *T_irr_* (irreversibility temperature), and *T_SRT_* (spin-reorientation transition temperature). 

In the ZFC *M*(*T*) curve, a notable rise in magnetization occurs at lower temperatures, with its magnitude greatly influenced by the intensity of the probe field. This rise features an inflection point (*T_f_*), which is identifiable as the lower kink in the curve. Following this, there is a gradual evolution up to 225 K, marked as *T_SRT_*, which is indicative of a spin-reorientation state wherein the in-plane magnetization transforms into perpendicular magnetization, as discussed elsewhere [4]. Subsequently, as the temperature increases further, magnetization steadily decreases, reaching its minimum value at 300 K. On the other hand, the FC *M*(*T*) curve demonstrates behaviour relatively unaffected by temperature and probe fields, eventually converging with the ZFC curve at the irreversibility temperature (*T_irr_*). Beyond the *T_SRT_*, the FC curve maintains a consistent decline, aligning with the trajectory of the ZFC curve up to 300 K. Notably, both *T_f_* and *T_irr_* exhibit significant shifts to higher temperature ranges under weaker applied fields, whereas *T_SRT_* remains unaffected by the strength of the probe field.

Examining the ZFC *M*(*T*) curve reveals three distinct regions: (I) a magnetization increase from 5 K to *T_irr_*, (II) a region of nearly constant magnetization (*T_irr_–T_SRT_*), and (III) a subsequent decrease from *T_SRT_* to 300 K. Remarkably, magnetization in the higher temperature region (*T_SRT_*–300 K) remains unaffected by variations in the applied probe field. In contrast, the other two regions exhibit a clear probe field dependence, with ZFC-FC curves mostly overlapping for stronger magnetic fields. This observation underscores the film’s dynamic magnetic response to varying probe fields and elucidates the complex interplay of magnetic transitions governing its behavior. Particularly, the thermal activation behavior of *H_C_*, as shown in Figure 2, closely correlates with the thermal characteristics observed in the ZFC magnetization curves at a lower temperature range. Analysing both *H_C_* and ZFC-FC magnetization curves across varying temperatures provides insights into the transformative states of ferromagnetic ordering within the amorphous matrix, leading to intriguing global magnetic behaviors as a function of temperature.

**Figure 4:**
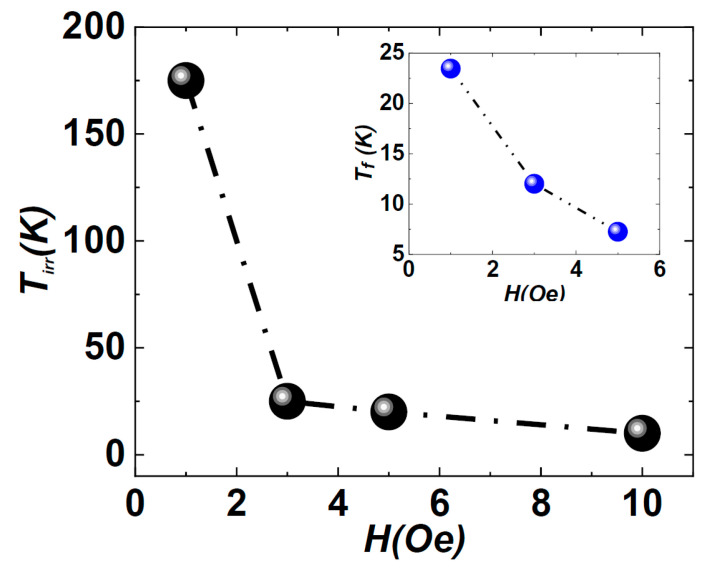
Irreversibility temperature (*T_irr_*) as a function of the probe field in the range of 1–10 Oe.

The pronounced splitting observed between the zero-field-cooled and field-cooled (ZFC-FC) magnetization curves serves as a sensitive indicator, emphasizing the contrast between randomly frozen magnetic moments and a fully aligned state, even under exceedingly weak magnetic fields [10,23]. The cooling process in the absence of an external magnetic field gives rise to a fascinating phenomenon, indicating the presence of multiple degenerate spin-configurations at low temperatures and weak probe fields [10]. This phenomenon is often observed in spin-glass-like systems, where spins experience frustration owing to disorder configurations and competing exchange interactions [23,24]. Within these systems, the thermal energy available at cryogenic temperatures is inadequate to surpass the energy barriers between these undefined states. Consequently, the system settles into random configurations without a preferred state. At such temperatures, the weak probe field remains insufficient to disrupt the symmetry of these established configurations, thereby allowing degenerate states to persist rather than forming a typical singular ground state. Within the temperature range from 5 K to *T_f_*, the irreversibility evident between ZFC-FC curves, mitigated by the application of stronger magnetic fields, indicates that the system enters a spin-glass state [24]. Figure 4 illustrates the evolution of the *T_irr_* with respect to the magnetic field, demonstrating a re-entrant spin-glass behavior [24].

The magnetization curves obtained at low temperatures in this study, under both ZFC and FC conditions, mirror the behavior observed in a broad range of materials. These include two-phase nanocrystalline Fe–B–Nb [8,10] and Fe–B–Zr [25] alloys produced by post-processing amorphous metal precursors, single-phase nanocrystalline iron particles [26], and magnetron-sputtered nanogranular Fe–Ag thin films [23,24,27]. In such systems, it is argued that the disordered arrangement of magnetic spins at the interfacial boundaries of nanocrystalline grains or on the surfaces of pure nanosized particles undergoes random freezing in the absence of a magnetic field [8,10,23,24,25,27]. This occurs by weak exchange coupling between Fe spins, resulting either from the small volume fraction of crystallites or due to the shielding effect from grain growth diffusion inhibitors surrounding nanograins in the amorphous matrix, leading to spin-glass-like behavior at cryogenic temperatures [10].

In our heterogeneous films, a situation analogous to that in nanocrystalline Fe–B–Nb alloys [8,10] can be assumed, where Fe nuclei are supposed to be contained by Nb diffusion inhibitors. Additionally, the infinitely small volume fraction of Fe nuclei, as previously discussed, is expected to be dispersed in a ferromagnetic matrix. Together, the shielding effect from diffusion inhibitors and the large separation between Fe nuclei due to a small volume fraction promote weak coupling similar to what is observed in Fe–B–Nb [8,10] and Fe–Ag [23,24,27] systems. This weak coupling causes the spins localized at the interfacial zones between nanosized grains and the amorphous matrix to undergo random freezing, marking the onset of a spin-glass-like state at near-cryogenic temperatures in weak/zero magnetic fields [23,24]. This transition introduces a unique aspect to the film’s behavior, as the randomly frozen spins diminish interparticle magnetic coupling between the Fe nuclei, prompting local anisotropy at lower temperatures to define the magnetic behaviours of the entire system [10,24]. The alignment of spins inside Fe nuclei may be subject to the influence of a randomly oriented easy axis, promoting a cluster-glass-like state within the system, thereby resulting in increased *H_C_* at cryogenic temperatures [8,10,23,24,27].

Expanding on this, our investigation into the magnetic hardening phenomenon within the extended Random Anisotropy Model (RAM) framework reveals the critical importance of examining the interfacial region between Fe nuclei and the amorphous matrix. This interface offers a unique prospect where the interfacial surface exhibits a temperature-dependent dynamic magnetic ordering state, acting as a barrier to magnetic coupling between Fe nuclei. Consequently, this redefines the fascinating magnetic reversal behaviors, such as magnetic hardening, observed in our system.

## 4. Conclusions

In conclusion, our investigation probed the low-temperature magnetic properties of Fe–Ni–B–Nb thin films, providing significant insights into the complex interplay between heterogeneous amorphous structure and magnetic dynamics at lower temperatures. Analysis of the film’s temperature-dependent coercivity response revealed a dynamic behavior, particularly notable at cryogenic temperatures. The unexpected occurrence of “magnetic hardening” below 25 K provoked a comprehensive exploration into magnetization dynamics, leading to exploration of the underlying mechanisms beyond the conventional Random Anisotropy Model (RAM) approach. This expanded investigation has yielded valuable insights into the complex magnetic interactions within a heterogeneous thin film material. The temperature-dependent magnetization response to varying probe fields has displayed dynamic transformations, notably at the spin-freezing temperature (*T_f_*) and the irreversibility temperature (*T_irr_*), confirming the system’s transition into a spin-glass state at low temperatures. The manifestation of spin-glass-like behavior underscores the films’ distinct magnetic response and poses challenges to direct interaction between nanoscale magnetic entities, thereby escalating magnetic hardening at cryogenic temperatures.

## Data Availability

Data is contained within the article.

## References

[1] Zhao S.Z., Li J.H., Liu B.X., Kimizaki H., Shinkai S., Sasaki K., Sharma P., Kaushik N., Kimura H., Saotome Y. (2007). Nano-Fabrication with Metallic Glass—An Exotic Material for Nano-Electromechanicalsystems. Nanotechnology.

[2] Mathúna C.Ó., Wang N., Kulkarni S., Roy S. (2012). Review of Integrated Magnetics for Power Supply on Chip (PwrSoC). IEEE Trans. Power Electron..

[3] Masood A., Belova L., Ström V. (2023). Magnetization Dynamics and Spin-Glass-like Origins of Exchange-Bias in Fe-B-Nb Thin Films. J. Appl. Phys..

[4] Masood A., Belova L., Strom V. (2024). Magnetic Anisotropy in Heterogeneous Amorphous Thin Films: Insights from Thickness and Temperature-Driven Spin-Reorientation. J. Phys. D Appl. Phys..

[5] Cronin D., Lordan D., Wei G., McCloskey P., Mathúna C.O., Masood A. (2020). Soft Magnetic Nanocomposite CoZrTaB-SiO2thin Films for High-Frequency Applications. J. Appl. Phys..

[6] Gemma R., Baben M.T., Pundt A., Kapaklis V., Hjörvarsson B. (2020). The Impact of Nanoscale Compositional Variation on the Properties of Amorphous Alloys. Sci. Rep..

[7] Coïsson M., Celegato F., Olivetti E., Tiberto P., Vinai F., Baricco M. (2008). Stripe Domains and Spin Reorientation Transition in Fe78 B13 Si9 Thin Films Produced by Rf Sputtering. J. Appl. Phys..

[8] Škorvánek I., Kováč J., Kötzler J. (2003). Nanocrystalline Soft Magnetic Materials: Intergrain Coupling and Spin Freezing Effects. Phys. Status Solidi.

[9] Suzuki K., Cadogan J. (1998). Random Magnetocrystalline Anisotropy in Two-Phase Nanocrystalline Systems. Phys. Rev. B.

[10] Škorvánek I., Skwirblies S., Kötzler J. (2001). Magnetic Hardening and Spin-Glass Phenomena in Nanocrystalline FeNbB at Low Temperatures. Phys. Rev. B-Condens. Matter Mater. Phys..

[11] Herzer G. (1990). Grain Size Dependence of Coercivity and Permeability in Nanocrystalline Ferromagnets. IEEE Trans. Magn..

[12] Herzer G. (1989). Grain Structure and Magnetism of Nanocrystalline Ferromagnets. IEEE Trans. Magn..

[13] Vázquez M., Marin P., Davies H.A., Olofinjana A.O. (1994). Magnetic Hardening of FeSiBCuNb Ribbons and Wires during the First Stage of Crystallization to a Nanophase Structure. Appl. Phys. Lett..

[14] Willard M.A., Laughlin D.E., McHenry M.E., Thoma D., Sickafus K., Cross J.O., Harris V.G. (1998). Structure and Magnetic Properties of (Fe0.5Co0.5)88Zr7B4Cu1 Nanocrystalline Alloys. J. Appl. Phys..

[15] Yoshizawa Y., Oguma S., Yamauchi K. (1988). New Fe-based Soft Magnetic Alloys Composed of Ultrafine Grain Structure. J. Appl. Phys..

[16] Hernando A., Marín P., Vázquez M., Herzer G. (1998). Thermal Dependence of Coercivity in Magnetic Nanostructures. J. Magn. Magn. Mater..

[17] Masood A., Biswas A., Ström V., Belova L., Ågren J., Rao K.V. (2011). The Effect of Ni-Substitution on Physical Properties of Fe 72-XB 24Nb 4Ni x Bulk Metallic Glassy Alloys. Mater. Res. Soc. Symp. Proc..

[18] Cronin D., Hardiman M., Lordan D., Wei G., McCloskey P., Oʹ Mathúna C., Masood A. (2021). Quantification of Residual Stress Governing the Spin-Reorientation Transition (SRT) in Amorphous Magnetic Thin Films. J. Magn. Magn. Mater..

[19] Chen N., Louzguine-Luzgin D.V., Yao K. (2017). A New Class of Non-Crystalline Materials: Nanogranular Metallic Glasses. J. Alloys Compd..

[20] Masood A., Belova L., Ström V. (2020). On the Correlation between Glass Forming Ability (GFA) and Soft Magnetism of Ni-Substituted Fe-Based Metallic Glassy Alloys. J. Magn. Magn. Mater..

[21] Alben R., Becker J.J., Chi M.C. (1978). Random Anisotropy in Amorphous Ferromagnets. J. Appl. Phys..

[22] Škorvánek I., Grössinger R. (2001). Low-Temperature Magnetic Hardening and Exchange Anisotropy in Nanocrystalline FeNbCrBCu Alloys. J. Magn. Magn. Mater..

[23] Alonso J., Fdez-Gubieda M.L., Barandiarán J.M., Svalov A., Fernández Barquín L., Alba Venero D., Orue I. (2010). Crossover from Superspin Glass to Superferromagnet in Fe_x_Ag_100−x_ Nanostructured Thin Films (20 ≤ x ≤ 50). Phys. Rev. B-Condens. Matter Mater. Phys..

[24] Alonso J., Fdez-Gubieda M.L., Sarmiento G., Chaboy J., Boada R., García Prieto A., Haskel D., Laguna-Marco M.A., Lang J.C., Meneghini C. (2012). Interfacial Magnetic Coupling between Fe Nanoparticles in Fe-Ag Granular Alloys. Nanotechnology.

[25] Garitaonandia J.S., Gorria P., Barquín L.F., Barandiarán J.M. (2000). Low-Temperature Magnetic Properties of Fe Nanograins in an Amorphous Fe-Zr-B Matrix. Phys. Rev. B.

[26] Bonetti E., Bianco L.D., Fiorani D., Rinaldi D., Caciuffo R., Hernando A. (1999). Disordered Magnetism at the Grain Boundary of Pure Nanocrystalline Iron. Phys. Rev. Lett..

[27] Alonso J., Fdez-Gubieda M.L., Fernández Barquín L., De Pedro I., Barandiarán J.M., Orue I., Svalov A., Sarmiento G. (2009). Collective Magnetic Behaviors of Fe-Ag Nanostructured Thin Films above the Percolation Limit. J. Appl. Phys..

